# Safe Administration of Carbon Nanotubes by Intravenous Pathway in BALB/c Mice

**DOI:** 10.3390/nano10020400

**Published:** 2020-02-24

**Authors:** José Jesús Guzmán-Mendoza, Silvia Lorena Montes-Fonseca, Ernesto Ramos-Martínez, Carmen González-Horta, Pilar del Carmen Hernández-Rodríguez, Erasmo Orrantia-Borunda, David Chávez-Flores, Blanca Sánchez-Ramírez

**Affiliations:** 1 Facultad de Ciencias Químicas, Universidad Autónoma de Chihuahua. Circuito No. 1 Campus Universitario II, Chihuahua CP 31125, Mexico; jjguzman@uach.mx (J.J.G.-M.); carmengonzalez@uach.mx (C.G.-H.); dchavezf@uach.mx (D.C.-F.); 2 Instituto Tecnológico de Monterrey Campus Chihuahua, Heroico Colegio Militar 4700, Col. Nombre de Dios, Chihuahua CP 31300, Mexico; silvialorena.montes@tec.mx; 3 Departamento de Anatomía Patológica del Hospital Ángeles Chihuahua. Av. Hacienda del Valle No. 7120, Chihuahua CP 31217, Mexico; eramos48@prodigy.net.mx; 4 Centro de Investigación en Materiales Avanzados (CIMAV), Miguel de Cervantes 120, Complejo Industrial Chihuahua, Chihuahua CP 31136, Mexico; erasmo.orrantia@cimav.edu.mx

**Keywords:** carbon nanotubes, toxicity, mice, renal damage, lung damage, nanotoxicology

## Abstract

Carbon nanotubes (CNTs) are nanomaterials with multiple possible uses as drug carriers or in nanovaccine development. However, the toxicity of CNTs administered intravenously in in vivo models has not been fully described to date. This work aimed to evaluate the toxic effect of pristine multi-walled CNTs (UP-CNTs), purified (P-CNTs), or CNTs functionalized with fluorescein isothiocyanate (FITC-CNTs) administered by intravenous injection in BALB/c mice. Biochemical and histopathological parameters were analyzed at 1, 14, 29, and 60 days post-exposure. Pristine CNTs were the most toxic nanoparticles in comparison with P-CNTs or FITC-CNTs, increasing serum AST (≈ 180%), ALT (≈ 300%), and LDH (≈ 200%) levels at one day post-exposure. The urea/creatinine ratio suggested pre-renal injury at the 14th day accompanied of extensive lesions in kidneys, lungs, and liver. Biochemical and histological findings in mice exposed to P-CNTs had not significant differences compared to the controls. A lower toxic effect was detected in animals exposed to FITC-CNTs which was attributable to FITC toxicity. These results demonstrate that the purification process of CNTs reduces in vivo toxicity, and that toxicity in functionalized CNTs is dependent on the functionalized compound. Therefore, P-CNTs are postulated as potential candidates for safe biomedical applications using an intravenous pathway.

## 1. Introduction

Carbon nanotubes are nanomaterials of choice in biomedical fields due to their broad applications [1,2,3]. Discovered by Sumio Iijima in 1991 [4], CNTs are described as cylindrical nanoparticles composed of a single wall (SWCNT), or multiple walls (MWCNT) of enrolled graphene sheets [5], which consist of perfectly structured carbon atoms rings [6].

The chemistry of CNTs resulted from efforts to open, fill and functionalize the sidewalls of the nanotubes. The latter can modify many of their physicochemical properties such as solubility and cytotoxicity [7]. CNTs can be functionalized with many groups such as proteins, peptides, nucleic acids, and synthetic molecules. Functionalized CNTs (*f-*CNTs) have a broad range of biomedical uses, such as drug-delivery systems [8,9], biosensors to detect cellular tumors [10], radiotherapeutic agents for radiological use [11], support for neuronal growth [12], and nanovaccine production [13], among others [1].

However, the increasing use and mass production of CNTs has sparked concern about the safety of the population and their environmental impact, but the evidence of their in vitro and in vivo toxicity, remains contradictory [14].

Our research group has demonstrated that purification processes, functionalization, and the nature of protein functionalization are important factors to consider during in vitro *f-*CNT cytotoxicity studies [15,16,17]. In order to be used in nanomedicine applications such as nanovaccines or drug delivery among others, in vivo studies are required to produce safer nanoparticles. In vivo studies have been directed to inhalation risk assessment of CNTs [18], while, information about their toxicity by parenteral or intravenous administration, one of the main routes used for drug delivery [19,20,21,22], is scarce [23]. Some studies have shown that *f*-CNTs injected into the bloodstream persisted within liver, lung, and spleen and were eliminated through the kidney and bile duct with low toxic effects [1,24,25].

Guo et al. used water-soluble *f*-CNTs labeled with radioactive atoms dosed intraperitoneally in mice to study their distribution throughout an organism. They demonstrated that *f-*CNTs move easily among compartments and tissues of the body without toxic effects [26,27].

Studies have shown that when CNTs reach the lung during the inhalation process, they can cause toxic effects. These effects depend mainly on the size, followed by the length-diameter ratio, and the surface area of CNTs [28]. CNTs toxicity is attributed to their physicochemical properties because the toxic effect can be modulated or diminished when their structure, surface area, conglomeration and degreee of oxidation, functional groups on their surface, and synthesis process are modified [29,30,31,32]. Previous reports from our research group evaluated the cytotoxicity of different CNTs in the J774 macrophage cell line. The results showed a similar response in an in vitro model; pristine MWCNTs (UP-CNTs) showed high cytotoxicity in all doses tested (0.6, 0.06 and 0.006 mg/mL), whereas FITC-functionalized CNTs (FITC-CNTs) showed a dose dependent effect, and finally the purified CNTs (P-CNTs) showed toxicity only at high concentrations [16].

Toxicology and pharmacology are closely related when a new CNTs-based drug or nanovaccine is developed. In vivo studies are needed to know if these new CNTs-based systems will have harmful effects on a whole organism, and to ensure the safety of their use [33]. Due to this, the objective of this work was to analyze the toxic effects of UP-CNTs, P-CNTs and FITC-CNTs in an in vivo model through the analysis of biochemical and histopathological parameters.

## 2. Materials and Methods

### 2.1. Nanoparticles

Multi-walled carbon nanotubes (UP-CNTs) were synthesized by spray pyrolysis, using toluene and ferrocene as carbon source and catalyst, respectively [34]. The purification process was carried out by sonication for 48 h in a mixture of concentrated H_2_SO_4_ (90%)/HNO_3_ (70%), (J.T. Baker, Loughborough, Leicestershire, UK) 3:1 v/v to afford P-CNTs [17].

UP- and P-CNTs were characterized by scanning electronic microscopy and energy dispersive X-ray Spectroscopy (SEM/EDS), using a model JSM-5800 LV instrument (JEOL, Akishima, Tokyo, Japan) and Raman spectroscopy using a micro-Raman LabRAM HR system (Horiba Jobin Yvon, Edison, NJ, USA) coupled to a BX-4 microscope (Olympus, Miami, FL, USA) and Spectrum Gx (Perkin Elmer, Hopkinton, MA, USA). The laser line used to excite the sample was 632.8 nm, and all measurements were performed at room temperature. The carboxyl groups on the P-CNTs were measured by titration with NaHCO_3_ based on a method established by Hu et al. [35] and modified by Montes-Fonseca et al. [15] as follows: 0.1 g of P-NTC was stirred in 50 mL of 0.05 N NaHCO_3_ (Sigma-Aldrich, St. Louis, MO, USA) aqueous solution. The mixture was then filtered through a membrane (pore size of 0.45 μm) and washed with deionized water to remove the NaHCO_3_ residues. The combined filtrate and washings were added to 50 mL of 0.05 N aqueous HCl (J.T. Baker, Loughborough, Leicestershire, UK) and boiled for 20 min to degas the CO_2_ of the solution. After cooling to room temperature, the excess of HCl was titrated with 0.05 N aqueous NaOH (Sigma-Aldrich, St. Louis, MO, USA) to reach a neutral pH 7.

### 2.2. Functionalization of P-CNTs

**Figure 1:**
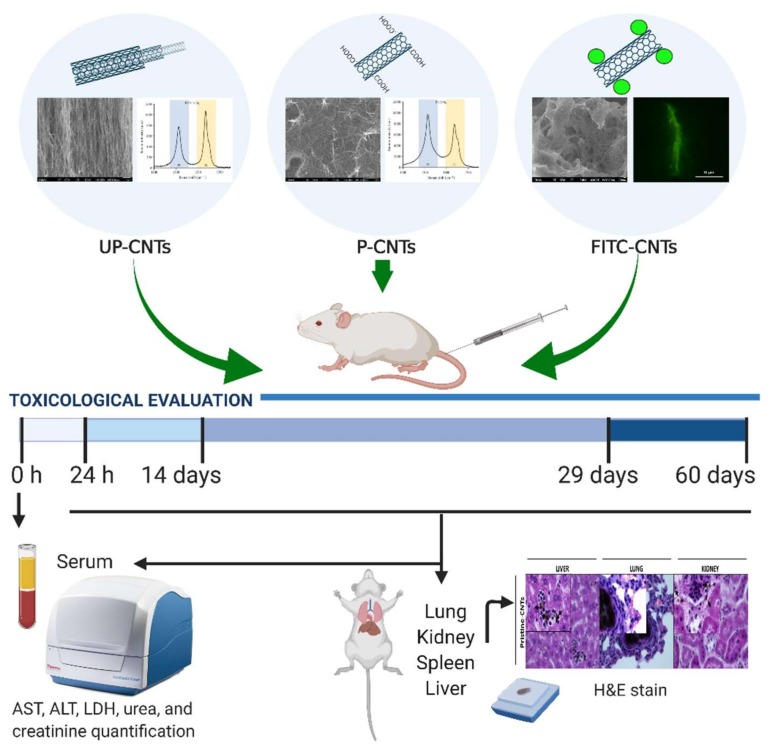
Timeline of exposure protocol for toxicological evaluation in BALB/c mice. The UP-CNTs and P-CNTs were characterized by SEM, and Raman spectroscopy; FITC functionalization was demonstrated by epi-fluorescence microscopy. Serum samples were obtained at 0, and 24 h, and later at 14, 29, and 60 days post inoculation of CNTs to determine the biochemical parameters. At the end of each time post inoculation, the animals were sacrificed, and the organs were dissected for histological observation.

P-CNTs were functionalized with fluorescein isothiocyanate (FITC; Sigma-Aldrich, St. Louis, MO, USA) by diimide-activated amidation (to obtain FITC-CNTs). Briefly, 35 mg of P-CNTs and 155 mg of 1-ethyl-3-(3-dimethylaminopropyl) carbodiimide (Sigma-Aldrich, St. Louis, MO, USA) were added to 25 mL of 0.1 M phosphate buffer pH 7.5. The suspension was sonicated for 2 h, and 350 mg of FITC were added and mixed for 24 h at room temperature (25 °C). Next, the suspension was centrifuged for 10 min at 7800 rpm. FITC-CNTs were recovered by filtration through a 0.45 µm pore size membrane filter and then were suspended in 2 mL of phosphate buffer pH 7.5. The functionalization was confirmed by epi-fluorescence microscopy (Figure 1, inset).

### 2.3. Animals and CNTs Administration

Male and female BALB/c mice (3 months age) weighing 30–35 g were provided by the farm of the Chemical Sciences Faculty; the Local Ethical Committee for Animal Research approved the experimental protocol (Reg. 001-2011). The animals were housed in cages (four mice per box) and maintained at 25 °C, 50% relative humidity with 12 h light/dark cycles (Figure 1). After acclimation, 64 mice were randomly divided into 16 groups exposed to a single dose of: 0.2% Pluronic F-127 (Sigma-Aldrich, St. Louis, MO, USA) as vehicle control, UP-CNTs, P-CNTs and FITC-CNTs. Suspensions of UP-, P-, or FITC-CNTs were prepared in sterile phosphate buffer pH 7.5 containing 0.2% of Pluronic F-127 as disperser solution [36,37], sonicating for homogeneous suspension before their use. A volume of 200 µL containing 2 mg/kg of body weight of each CNTs was injected by intravenous via (i.v), using the tail vein. The dose was based on a predictive Bayesian dose-response assessment for evaluating the toxicity of CNTs [38]. At 1, 14, 29, and 60 days post exposure animals were anesthetized with sodium pentobarbital Sedalpharma® (Pets Pharma, Edo. de Mex., México) and sacrificed by exsanguination.

### 2.4. Histopathological Analysis

Liver, lungs, kidneys, and spleen were dissected, weighed, and fixed in 4% buffered paraformaldehyde solution (Sigma-Aldrich, St. Louis, MO, USA). Paraffin-embedded sections (6 μm thick) were prepared in a RM2125 RTS microtome (Leica, Buffalo Grove, IL, USA) and stained with hematoxylin and eosin (H&E), to assess for histological damage and CNTs accumulation (Olympus BX41) (Olympus, Miami, FL, USA).

### 2.5. Biochemical Analysis

As damage biomarkers, aspartate aminotransferase (AST; EC 2.6.1.1), alanine aminotransferase (ALT; EC 2.6.1.2), lactate dehydrogenase (LDH; EC 1.1.1.27), urea and creatinine, were quantified. After anesthesia, samples of retro-orbital venous blood were obtained before and at the end of the post-exposure time. Serum was obtained by centrifugation at 3500 rpm (4 °C) using a 5804 R centrifuge (Eppendorf, Hauppauge, NY, USA). Enzymatic activities measurments were performed immediately after the serum was obtained; normal and pathologic control serum (Serodos® and Serodos Plus®; HUMAN, Biochemica und Diagnostica GmbH; Wiesbaden, Germany) were used in all determinations. All determinations were done in triplicate using HUMAN kits (Biochemica und Diagnostica GmbH) and following the manufacturer’s protocols adapted for microplates, using a Varioskan Flash reader (Thermo Scientific, Waltham, MA, USA).

Urea quantification was carried out using the kit liquiUV (REF: 10521). Briefly, 1 µL of each serum sample was added to 100 µL of buffer #1 in a 96 well plate. Controls with 1 µL of urea standard (80 mg/dL) was added to 100 µL of buffer #1 in another well. After that, 25 µL of buffer #2 was added to samples or standard and stirred. Absorbance was determined at 340 nm at 37 °C.

Auto-CREATININE liquicolor (REF: 10052) was used for the creatinine quantification. Briefly, 5 µL of each serum sample was added to 50 µL of buffer #1 in a 96 well plate. Controls with 5 µL of creatinine standard (80 mg/dL) was added to 50 µL of buffer #1 in another well. After that, 25 µL of buffer #2 was added to samples or standard and stirred. Absorbance was determined at 510 nm at 37 °C.

LDH activity was determined using a kit LDH SCE mod. liquiUV Humazym Test (REF: 12014). In brief, 1 µL of each serum sample was added to 100 µL of reactive #1 in a 96 well plate. After that, 25 µL of reactive #2 was added to each well, stirred, and absorbance was measured at 340 nm.

ALT activity was determined using the kit GPT (ALAT) IFCC mod. liquiUV Humazym Test (REF: 12012). In brief, 10 µL of each serum sample was added to 50 µL of reactive #1 in a 96 well plate. The plate was stirred and incubated by 5 min at 25 °C. After that, 12.5 µL of reactive #2 was added to samples, stirred, and absorbance was measured at 340 nm.

AST activity was determined using the kit GOT (ASAT) IFCC mod. liquiUV Humazym Test (REF:12011). In brief, 10 µL of each serum sample was added to 50 µL of reactive #1. The plate was stirred and incubated by 5 min at 25 °C. After that, 12.5 µL of reactive #2 was added to samples, stirred, and absorbance was measured at 340 nm.

### 2.6. Statistical Analysis

Data in graphs are expressed as the increase or decrease in percentage of enzymatic activity or metabolite concentration after the CNTs administration taking as 100% the basal enzymatic activity or the metabolite concentration in the same animal before the CNTs administration. Data were analyzed using the MINITAB statistical software v. 17 (Minitab Inc.; State College, PA, USA), mean comparisons were performed using ANOVA and Fisher LSD method.

### 2.7. Creative Images

Creative images were performed using BioRender.com.

## 3. Results

### 3.1. Nanoparticles Characterization

The UP-CNTs and P-CNTs used were previously characterized by our research group [15]. UP-CNTs had on average 20–40 nm in diameter and 30 μm in length. P-CNTs showed a considerable decrease in length to <1 μm and a 7% increase of COOH groups according to the titration assay, and better dispersion in water. The Raman spectra provided information about the structure of the CNTs. This spectrum usually shows two bands at 1338 and 1600 cm^–1^, namely D and G, respectively. G-band is a characteristic feature of the graphite layers and corresponds to the tangential vibration of the carbon atoms, while the D-band is indicative of the presence of defects in the wall. UP-CNTs showed a G-band intensity greater (1090) that D-band (790), while P-NTCs showed a greater D peak intensity that G-band (9800 and 7950, respectively) (Figure 1, inset). These data indicate that P-CNTs have more defects in their wall due to the purification process, where CNTs were shortened and oxidized for their future functionalization. Functionalization of CNTs with FITC was evidenced by epifluorescence microscopy (Figure 1, inset).

### 3.2. Morphological Findings

As shown in Table 1, a significant increase in body weight was detected in animals of the group exposed to UP-CNTs and P-CNTs only at 14 days postexposure. When the weight gain was analyzed, a significantly lower weight gain was detected only in the group exposed to UP-CNTs at 29 days postexposure; weight gain was recovered in animals exposed to that nanoparticles at 60 days (Table 1).

**Figure 2:**
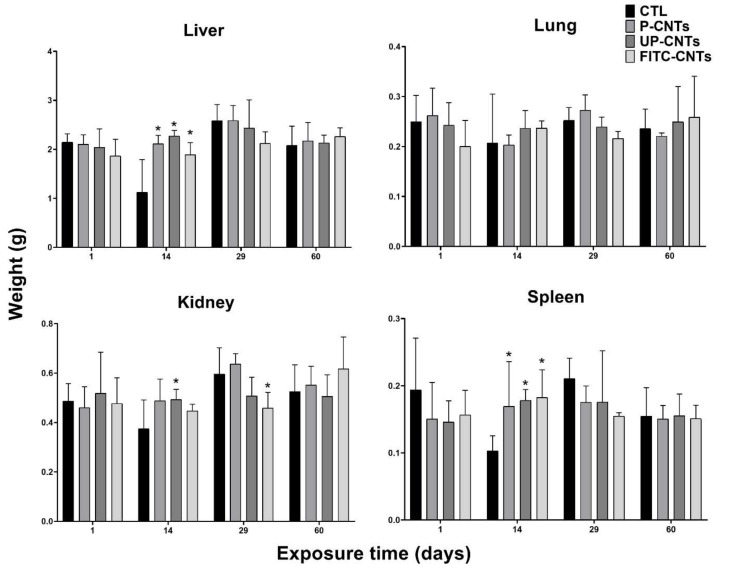
Organ weights (g) from mice exposed to different CNTs at different exposure times. Each graph shows the data of the indicated organ, the bars represent the mean ± SD obtained for each group (*n* = 4). CTL, vehicle control (0.2% Pluronic F127 in sterile phosphate buffer). * indicates significant differences vs the control group (α= 0.05). Data were analyzed by Dunnett’s test.

Regarding organs, a significant increase in liver weight was detected at 14 days post-exposure in groups exposed to CNTs, independently of functionalization (Figure 2); no significant changes were detected at any other post-exposure times.

**Figure 3:**
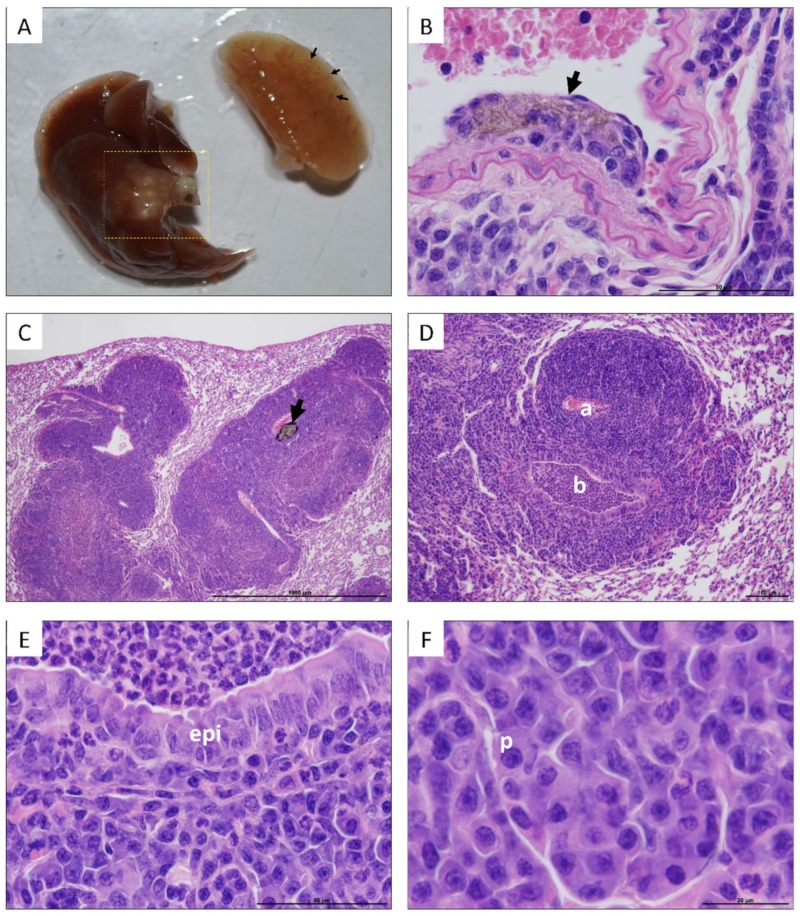
Macroscopic and microscopic lesions observed in lungs of mice exposed to UP-CNTs 60 days after exposure. **A**, Macroscopic aspect of lungs from a mouse exposed to UP-CNTs; the black arrows indicate the accumulation of nanoparticles mainly in the peripheral areas. The dotted line on the left lung indicates a tumor growth lesion. Microphotographs show: **B**, UP-CNTs trapped under endothelium (arrow) found in the right lung (magnification 60×); **C**, general aspect of lesions detected in the left lung, the arrow indicates the accumulation of UP-CNTs (arrow, magnification 4X); **D**, malignant lymphoproliferative lesion of peribronchial and perivascular localization, artery (**a**) is surrounded by several layers of large lymphocytes, bronchiole (**b**) looks obstructed by neutrophils (magnification 10×); **E**, the bronchiole epithelium (**epi**) in contact with neutrophils inside luminal space (magnification 60×), and; **F**, an area with large lymphocytes with abnormal nuclei (plasmocyte, **p**) (magnification 100×).

In the case of the lungs, no significant changes in weight were detected based on the exposure time or the type of CNTs used (Figure 2). Notwithstanding, lungs of some mice exposed to UP-CNTs at 60 days post-inoculation had a visible decrease in size and were mainly affected in the right lung (Figure 3A) and which was probably caused by vascular obstruction due to nanoparticles (Figure 3B). Additionally, left lung presented a malignant lymphoproliferative process localized mainly at peribronchial and perivascular localization (Figure 3C, D). A notable infiltrate with neutrophils and abnormal large lymphocytes (plasmacytoid cells) was detected surrounding the bronchioles (Figure 3E,F).

A significant increase in kidney weight was detected in mice exposed to UP-CNTs at 14 days, decreasing at 29 days post-exposure (Figure 2). In the case of mice exposed to FITC-CNTs, a significant decrease in kidney weight was observed only at 29 days post-exposure. Regarding spleen, a significant weight increase was perceived at 14 days post-exposure in all groups exposed to CNTs; however, the highest increase ocurred with FITC-CNTs (Figure 2).

Macroscopic findings showed that livers of mice exposed to UP-CNTs and FITC-CNTs presented changes in their color and appearance, showing a dark red coloration, and like the lungs of the same group, livers presented necrotic areas. On the other hand, the organs of mice exposed to P-CNTs showed normal characteristics, like those observed in the control group (data not shown).

**Figure 4:**
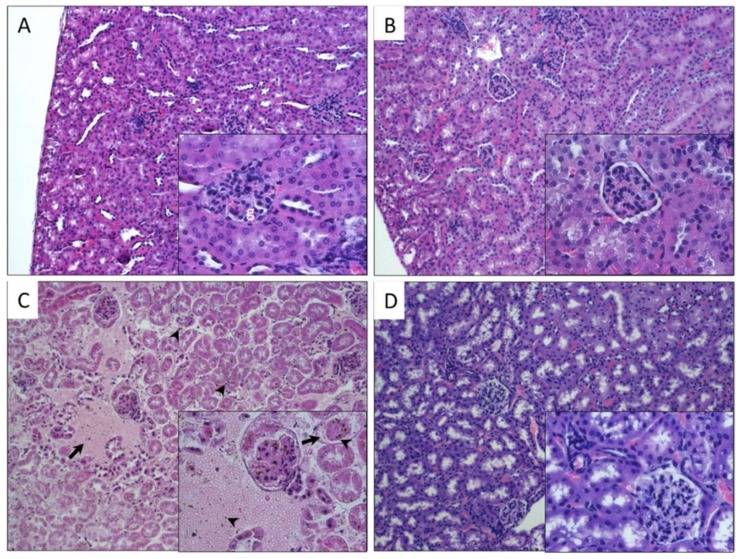
Histopathological findings in kidney of mice exposed to different types of CNTs. Microphotographs show kidney sections of mice exposed to: **A**, control group non exposed, the normal morphology of renal cortex (**g**, glomerulus); **B**, P-CNTs exposed group. **C**, UP-CNTs exposed group, hemorrhagic areas (arrows) and small spherical bodies trapped in glomeruli and damaged areas (arrowheads) that corresponds to UP-CNTs. **D**, FITC-CNTs exposed group. Microphotographs are representative of one experiment. Magnifications 10×. Insets show magnification at 40×. H&E staining.

Histological analysis of kidneys from mice exposed to different CNTs was compared with the morphology of the control group (Figure 4A). As shown in Figure 4B, the kidney of mice exposed to P-CNTs did not show alterations, glomeruli and tubules looked normal, and there was no accumulation of P-CNTs in this tissue. On the other hand, kidneys of mice exposed to UP-CNTs showed alterations in the renal tubules with the accumulation of UP-CNTs in the glomeruli (Figure 4C), and also presented infarction probably due to vascular obstruction that causes tissue necrosis, resulting in hypocellularity, and hemorrhagic areas with disruption of renal parenchyma. Regarding kidney of mice exposed to FITC-CNTs, they did not present important alterations (Figure 4D).

**Figure 5:**
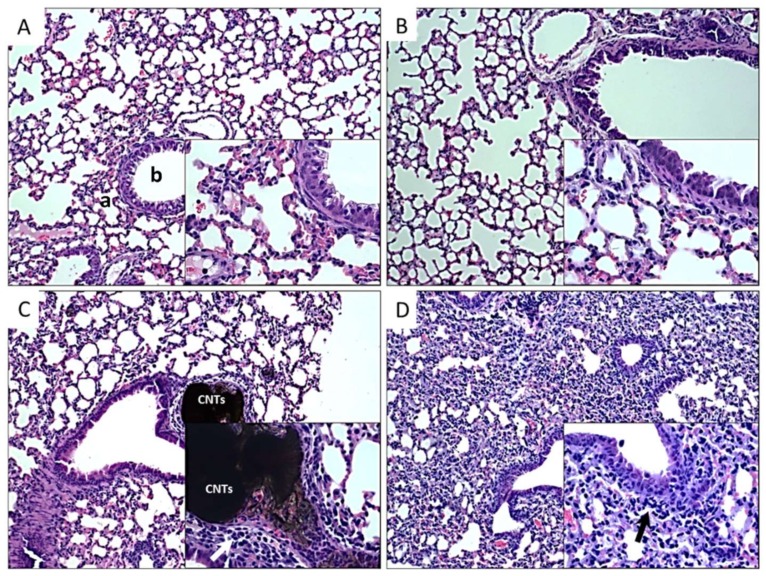
Histopathological findings in lungs of mice exposed to different types of CNTs. Microphotographs show lung sections of mice exposed to: **A**, control group non exposed, the normal morphology of bronchiole (**b**) and alveoli (**a**) is shown; **B**, P-CNTs exposed group. **C**, UP-CNTs exposed group, accumulation of CNTs in the artery, and inflammatory infiltrate in alveolar walls (arrow). **D**, FITC-CNTs exposed group, inflammatory infiltrate (arrow), of note is the decrease of the alveolar sacs in the lung. Microphotographs are representative of one experiment. Magnification 10X. Insets show magnification at 40×. H&E staining.

As shown in Figure 5A,B, lungs from control and P-CNTs exposed group looked quite similar, with bronchioles and alveolar walls free of inflammatory cells, and no significant detectable damage; moreover, they did not show accumulation or persistence of these nanoparticles even after prolonged exposure (60 days). In the case of lungs of mice exposed to UP-CNTs, constriction of alveoli and bronchiole was observed, accompanied by multiple inflammatory cells (Figure 5C). Also, accumulation of nanoparticles was found obstructing small bronchioles (see insert in Figure 5C). Similar damage was observed in lung sections of mice exposed to FITC-CNTs, where alveoli were diminished, showing an inflammatory process with the absence of granulomatous foci and CNTs accumulations (Figure 5D). The cellular infiltrate was composed mainly of neutrophil granulocytes and macrophages.

### 3.3. Biochemical Findings

Enzymatic activity of AST, ALT, and LDH, and the concentration of urea and creatinine in the serum of all animal exposed to nanoparticles and in the control group, was determined as toxicity biomarkers.

**Figure 6:**
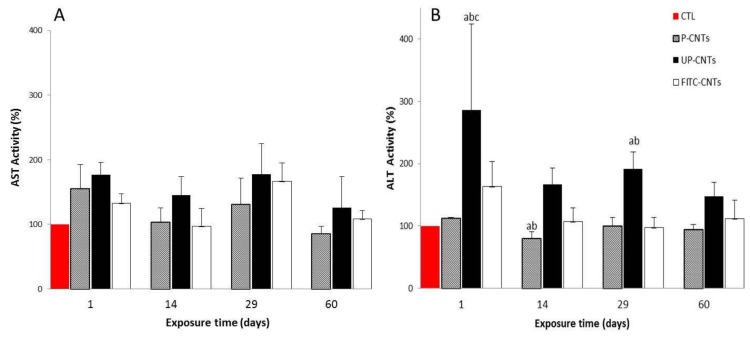
Effect of exposure with different CNTs on the relative activity of AST and ALT. Relative activity of serum aminotransferases in mice exposed to different CNTs at 1, 14, 29, and 60 days post exposure. (**A**) AST and (**B**) ALT activities. Each bar represents the mean ± SE of each group (*n* = 4). a: difference with respect to the control (*p* < 0.05). b: difference among treatments (*p* < 0.05), and c: difference among times post exposure (*p* < 0.05).

Mice exposed to UP-CNTs showed highest values of AST at all times tested; also the group exposed to P-CNTs showed an increase in AST activity only at 24 h post exposure, followed by a decrease of AST activity at 14 and 60 days (Figure 6A). The group exposed to FITC-CNTs showed a similar behavior that the mice exposed to P-CNTs (Figure 6A). No statistically significant differences in AST activity were observed in any treatment compared with the levels of the control group.

Mice exposed to UP-CNTs showed a significant increase in ALT values at 24 h and 29 days post- exposure (Figure 6B). A minor (non-significant) increase was detected in ALT activity in mice exposed to FITC-CNTs at 24 h post exposure. In mice exposed to P-CNTs a significant decrease was detected at 14 days post-exposure, however, this decrease has no clinical relevance.

**Figure 7:**
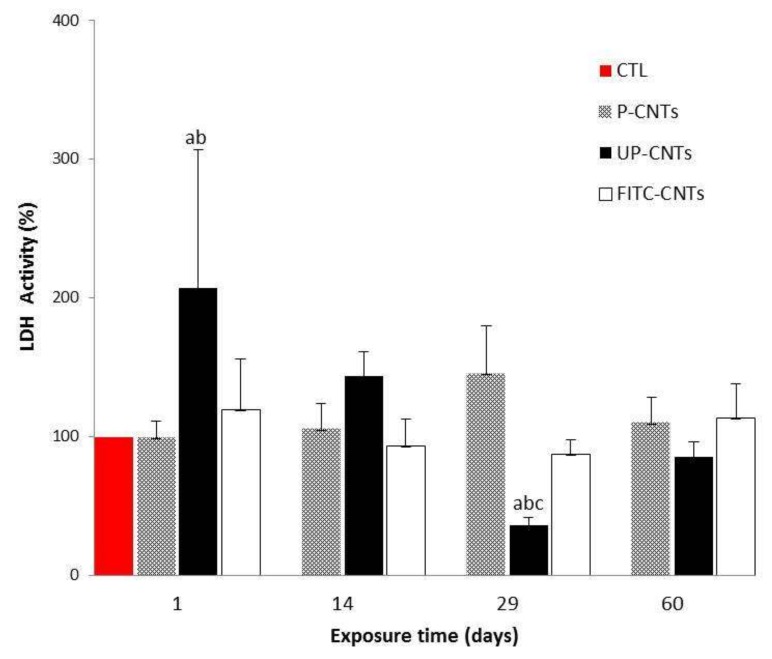
Effect of exposure with different CNTs on the relative activity of LDH. Relative activities of serum LDH in mice exposed to different CNTs at 1, 14, 29, and 60 days post exposure. Each bar represents the mean ± SE of each group (*n* = 4). a: difference with respect to the control without stimulus (*p* < 0.05). b: difference between treatments (*p* < 0.05), and c: difference between times post exposure (*p* < 0.05).

On the other hand, LDH activity is an important toxicity parameter and is related to pulmonary and muscle injuries. LDH activity of groups exposed to CNTs is shown in Figure 7. Again the group exposed to UP-CNTs showed a significant increase at 24 h post-exposure, decreasing significantly at 29 days, and reaching the control group values at 60 days. FITC-CNTs and P-CNTs did not show differences compared with the control.

**Figure 8:**
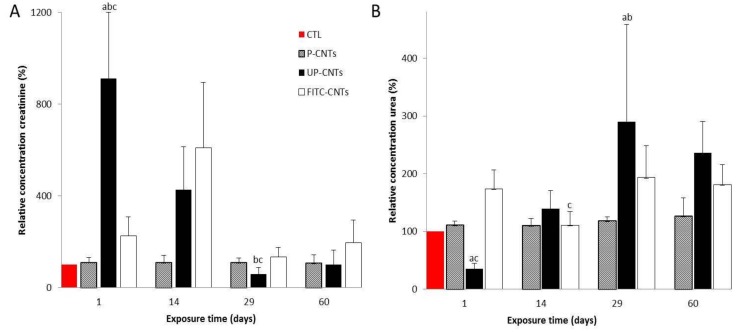
Effect of exposure to different CNTs on levels of urea and creatinine. Graphics show relative concentration of **A,** urea and **B,** creatinine, at 1, 14, 29, and 60 days post exposure. Each bar represents the mean ± SE of each group (*n* = 4). a: difference with respect to the control without stimulus (*p* < 0.05). b: difference among treatments (*p* < 0.05), and c: difference among times post exposure (*p* < 0.05).

Creatinine determinations (Figure 8A) showed an increase in the concentration of this biomarker at 24 h and 14 days post-exposure for the mice exposed to UP-CNTs. No significant differences were detected in mice exposed to P-CNTs or FITC-CNTs. Conversely, urea levels presented a significant decrease in mice exposed to UP-CNTs at 24 h post-exposure (Figure 8B). Levels in this group had a significant increase at 29 days and decreased at 60 days post-exposure. No significant changes were detected in mice from groups exposed to P-CNTs or FITC-CNTs. Results from UP-CNTs exposed group revealed renal damage.

The urea/creatinine ratio (Table 2) allowed us to determine the type of renal alteration. After 24 h, the urea/creatinine ratio was very low in the group exposed to UP-CNTs, indicating a low production of urea, a condition related to liver damage. This result is in agreement with the elevation of the hepatic enzymes (AST and ALT) in the same group. At 14, 29 and 60 days the urea/creatinine ratio increased, suggesting a pre-renal condition, congestive heart failure or hemorrhage. In the group exposed to FITC-CNTs, a non-significant increase in urea levels was observed at 24 h and 29 days; this may be associated with renal damage attributed to FITC [39].

## 4. Discussion

The toxicity of nanomaterials are strictly related with impurities that remain, in this case, on CNTs’ surfaces and that are the result of catalysts or reactants used in their synthesis. As well as in in vitro studies reported by our group [15,16], P-CNTs were the less toxic nanomaterials. Previous reports have demonstrated that purification using an acid treatment reduces CNTs’ toxicity due to the elimination of ferrocene and to the increase in the nanomaterials’ solubility. In addition, acid treatment introduces carboxyl groups on CNTs’ surfaces which allow the interaction with plasma proteins and contributes to their interaction with cells [40]. Corona proteins could be participating in the decrease of toxicity and facilitating the interaction with immune cells.

On the other hand, Shvedova et al. [41] detected foci of granulomatous inflammation in lungs of mice exposed to UP-CNTs by the inhalation pathway. These observations suggest that UP-CNTs must have a chemical preference for lungs which could be related to the presence of pulmonary lipophilic surfactant [42] since, independently of the exposure pathway, UP-CNT tend to reach and stay in lungs. This is a relevant point, due to the fact that these nanoparticles have preferences for some tissues and show target organs upon systemic exposure of animals to CNTs. In addition, the lymphoma growth observed at 60 days in the lung of a mouse, shows the importance of conducting studies at more prolonged post-exposure times. Pulmonary fibrosis observed in the group exposed to FITC-CNTs could be attributed to the FITC molecule. Christensen et al. [43] demonstrated that FITC induced pulmonary fibrosis in mice, with the presence of alveolar edema, eosinophilic exudate, hemorrhage, and inflammation. Similarly, our results agree with those reported by Ji et al. [44] who observed severe hepatotoxicity in mice exposed to UP-CNTs by i.v. pathway in a dose of 10 and 60 mg/kg of body weight, with irregular necrosis, infiltration of inflammatory cells, and hepatocyte lysis. These results suggest that the damage in the liver is the same even at low doses (2 mg/kg used in this study) (Appendix, Figure S1). Besides when CNTs were treated with acids (purification), the toxicity decreased in a similar way with the results that we are presenting in the groups exposed to P-CNTs (Figure 5B). Interestingly, exposure to UP-CNTs increased notoriously the cellularity in spleens (Appendix, Figure S2); this event might be related with the capacity of spleen to retain exogenous materials for body decontamination, and/or with the ability of macrophages to phagocyte and retains UP-CNTs. Besides, sequestration of UP-CNTs might probably be responsible for the increase in spleen tissue staining. A better interaction of purified and functionalized CNTs with plasma and extracellular proteins could improve the systemic distribution of nanomaterials and favor their use in biomedicine.

Serum AST activity is considered a less effective biomarker to detect liver damage, however, its increase could be related to pulmonary embolism, or congestive heart failure [45]. On the other hand, an increase in the ALT activity is strongly related to liver damage. After 24 h of exposure, an important increase of ALT activity was observed in mice exposed to the UP-CNTs in comparison with the control, followed by a decrease at 14, and 29 days post exposure (Figure 6B). Values of ALT for this group were higher than the values of other exposed groups. This increase could be due to the UP-CNTs behaving like a xenobiotic; the liver tries to transform and eliminate them, promoting their accumulation and causing liver damage. Increase in ALT levels in some cases could be related to heart disease or congestive heart failure; in this case the UP-CNTs i.v. administered could reach and agglomerate in heart producing damage and a consequent increase in the ALT activity. Similar results were reported by Meng et al. with an increase in the AST and ALT activity in mice exposed to UP-SWCNTs (4 mg/kg of body weight) by inhalation route at 16 days post-exposure [46]. Additionally, these authors found that mice exposed to purified-SWCNTs, folic acid, and chitosan-functionalized SWCNTs showed the lowest values in the activity of these enzymes in comparison with UP-SWCNTs. Taking together these results suggest that the liver damage caused by the CNTs is independent of the administration route and that these effects can be decreased by purification and functionalization of CNTs. Additionally, when analyzing the relationship between ALT and AST, it was observed that the ALT values of the group exposed to UP-CNT surpass, at all the times analyzed, the AST values, which is indicative of an inflammatory condition of the liver. Similar results were reported by Warheit et al. [47] after a single wall unpurified CNT administration (5 mg/kg of body weight) in rats. This administration produced mortality in the ~15% range in the SWCNT-instilled rats within 24 h post-instillation. This result suggests acute lung toxicity caused by the UP-CNTs, which we also observed.

## 5. Conclusions

Due to the great applications of CNTs in medical uses the evaluation of their toxicity is necessary. In this research, we focused on the in vivo toxicity of different multi-walled carbon nanotubes intravenously administered in a single dose, where we conclude that UP-CNTs have the highest toxicity, particularly due to accumulation in the kidneys and the lungs even at 60 days; moreover, they produced lung damage, tumor growth, hepatotoxicity, renal failure and could possibly induce heart failure. The less toxic CNTs were FITC-CNTs, followed by P-CNTs where we couldn’t observe any apparent toxic effect, or their accumulation. These results suggest that the purification process and the nature of the molecule functionalized are determining factors for in vivo toxicity of the CNTs here tested. In this study we postulate P-CNTs as potential candidates for biomedical applications using i.v. injection due that these nanoparticles did not show toxic effects or accumulation at the dose and time tested.

## Figures and Tables

**Table 1:** Body weight and weight gain (g) in mice exposed to different CNTs and at different exposure times.

Groups	24 h	14 days	29 days	60 days
Final body weight (g)				
CTL	35.7 ± 1.9	27.3 ± 6.8	42.2 ± 4.4	38.6 ± 3.9
UP-CNT	38.1 ± 5.7	38.5 ± 2.6 *	41.2 ± 2.8	40 ± 2.1
P-CNT	36.5 ± 5.6	37.8 ± 5.6 *	44.2 ± 4.3	38.7 ± 2.9
FITC-CNT	37.15 ± 4.7	33.3 ± 1.6	37.7 ± 1.8	37.2 ± 3.2
Body weight gain (g)				
CTL	–1.3 ± 0.9	–1.53 ± 4.2	7.9 ± 2.07	5.8 ± 1.5
UP-CNT	–1.1 ± 1.5	2.5 ± 1.08	2.12 ± 1.3 *	10.15 ± 3.4 *
P-CNT	–0.8 ± 1.7	1.6 ± 1.2	10 ± 2.04	9.2 ± 2.1
FITC-CNT	–1.8 ± 0.8	0.75 ± 2.6	7.2 ± 3.6	7.8 ± 3.6

Results are shown as media ± SD (*n* = 4). * Indicate significant difference vs the control group ( = 0.05). Data were analyzed by Dunnett’s test. CTL, vehicle control (0.2% Pluronic F127 in sterile phosphate buffer).

**Table 2:** Urea/creatinine ratio in the serum of the mice exposed to UP-CNTs at different exposure times.

Biomarker	Time Post Exposure (Days)			
	1	14	29	60
Urea (mg/dL)	16.08	75.95	77.72	87.52
Creatinine (mg/dL)	2.91	1.79	0.30	0.34
Urea/Creatinine Ratio *	**2.58**	**19.83**	**121.03**	**120.26**
Damage	Hepatic damage	Pre-renal kidney injury	Pre-renal/Hemorrhagic Status	

Results are shown as mean ± S.D. (*n* = 5). * Laboratory test handbook, 4th Ed, Lexi-Comp Inc.: Hudson, OH, USA, 1996; p. 199. Free Software: http://www.senefro.org/modules.php?name=nefrocalc&file=calculadoras&calc=nef_urecre.

## Data Availability

The datasets supporting the conclusions of this article are included within the article and its additional files.

## Supplementary Materials

The following are available online at https://www.mdpi.com/2079-4991/10/2/400/s1, Figure S1: Histopathological findings in livers from mice exposed to different types of CNTs. Microphotographs show liver sections of mice exposed to: A, control group non exposed, the normal morphology of liver parenchyma, a portal triad is shown in inset; B, P-CNTs exposed group. C, UP-CNTs exposed group, vacuolization of parenchyma (arrows) and accumulation of CNTs (arrowheads). D, FITC-CNTs exposed group, weak edema is observed. Microphotographs are representative of one experiment. Magnification 10×. Insets show magnification at 40×. H&E staining, Figure S2: Histopathological findings in spleens from mice exposed to different types of CNTs. Microphotographs show spleen sections of exposed mice: A, non-exposed control group, the normal morphology of spleen tissue; B, P-CNTs exposed group. C, UP-CNTs exposed group, some areas show an increase in cellularity with hyperchromasia, and at the bottom, there is a loss of cells with CNTs deposits. D, FITC-CNTs exposed group. Microphotographs are representative of one experiment. Magnification 10×. Insets show magnification at 40×. H&E staining. Table S1: Relative organ weights (%) of mice exposed to different CNTs at different exposure times.

## Abbreviations

ALT: alanine aminotransferase; AST: aspartate aminotransferase; CNTs: carbon nanotubes; CTL: control; *f*-CNTs: functionalized carbon nanotubes; FITC-CNTs: Fluorescein isothiocyanate functionalized carbon nanotubes; LDH: lactate dehydrogenase; MWCNT: multiple walls carbon nanotubes; P-CNTs: purified carbon nanotubes; SEM: scanning electronic microscopy; SWCNT: single wall carbon nanotubes; UP-CNTs: pristine multi-walled carbon nanotubes.
