# Supplementary material for: Safe Administration of Carbon Nanotubes by Intravenous Pathway in BALB/c Mice
Source: Nanomaterials (Basel). 2020 Feb 24;10(2):400. doi: 10.3390/nano10020400 (PMC7075304; doi:10.3390/nano10020400)
Supplement: Supplementary file 1 [file nanomaterials-10-00400-s001.pdf]

Supplementary

Safe Administration of Carbon Nanotubes by Intravenous Pathway in BALB/c Mice

Jesús Guzmán-Mendoza [1], Silvia Lorena Montes-Fonseca [2], Ernesto Ramos-Martínez [3], Carmen González-Horta [1], Pilar del Carmen Hernández-Rodríguez [1], Erasmo Orrantia-Borunda [4], David Chávez-Flores [1] and Blanca Sánchez-Ramírez [1,*]

[1] Facultad de Ciencias Químicas, Universidad Autónoma de Chihuahua. Circuito No. 1 Campus Universitario II, CP 31125 Chihuahua, México; jjguzman@uach.mx (J.G.-M.); carmengonzalez@uach.mx (C.G.-H.); pilar_hernandez@inclair.com (P.d.C.H.R.); dchavezf@uach.mx (D.C.-F.)

[2] Instituto Tecnológico de Monterrey Campus Chihuahua, Heroico Colegio Militar 4700, Col. Nombre de Dios, CP 31300. Chihuahua, México; silvialorena.montes@tec.mx

[3] Departamento de Anatomía Patológica del Hospital Ángeles Chihuahua. Av. Hacienda del Valle No. 7120, CP 31217. Chihuahua, México; eramos48@prodigy.net.mx

[4] Centro de Investigación en Materiales Avanzados (CIMAV), Miguel de Cervantes 120, Complejo Industrial Chihuahua, CP 31136. Chihuahua, México; erasmo.orrantia@cimav.edu.mx

* Corresponding author: bsanche@uach.mx; Tel.: +52-614-255-0177

Received: 31 January 2020; Accepted: 21 February 2020; Published: date

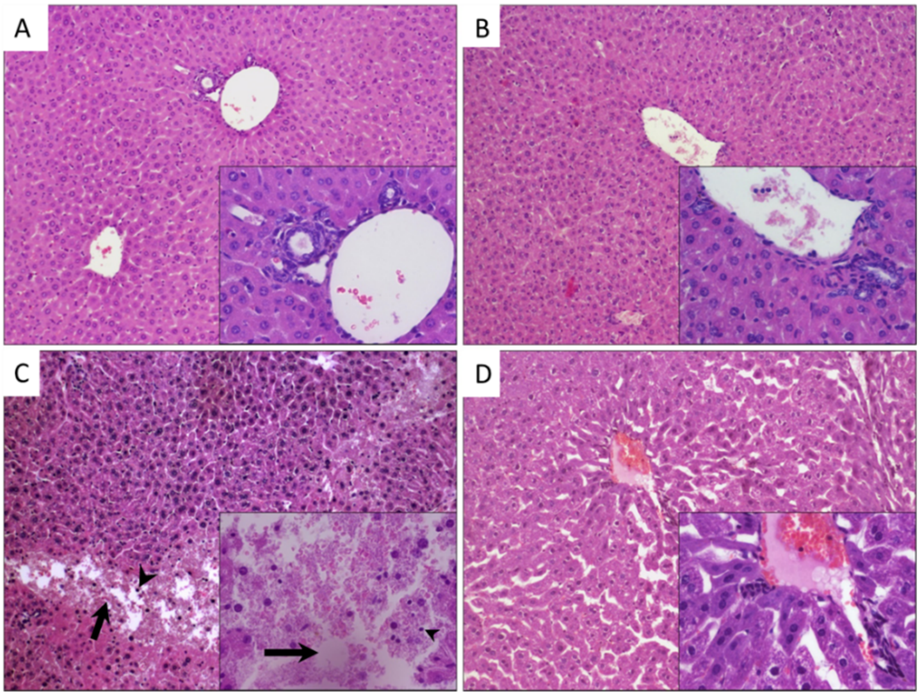

Figure S1. Histopathological findings in livers from mice exposed to different types of CNTs. Microphotographs show liver sections of mice exposed to: A, control group non exposed, the normal morphology of liver parenchyma, a portal triad is shown in inset; **B**, P-CNTs exposed group. **C**, UP-CNTs exposed group, vacuolization of parenchyma (arrows) and accumulation of CNTs (arrowheads). **D**, FITC-CNTs exposed group, weak edema is observed. Microphotographs are representative of one experiment. Magnification 10×. Insets show magnification at 40×. H&E staining.

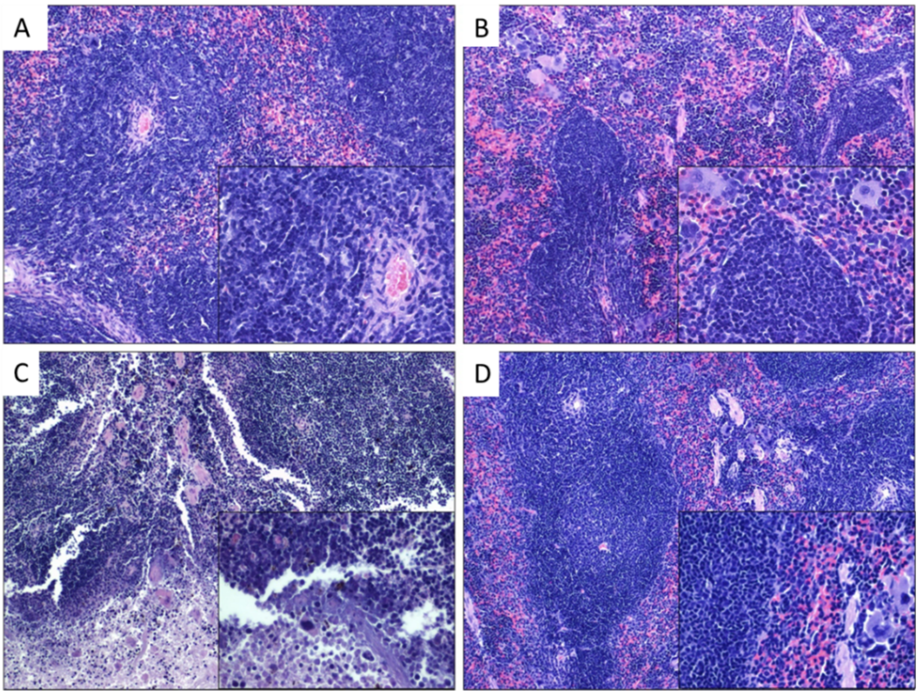

Figure S2. Histopathological findings in spleens from mice exposed to different types of CNTs. Microphotographs show spleen sections of exposed mice: **A**, non-exposed control group, the normal morphology of spleen tissue; **B**, P-CNTs exposed group. **C**, UP-CNTs exposed group, some areas show an increase in cellularity with hyperchromasia, and at the bottom, there is a loss of cells with CNTs deposits. **D**, FITC-CNTs exposed group. Microphotographs are representative of one experiment. Magnification 10×. Insets show magnification at 40×. H&E staining.

Table S1. Relative organ weights (%) of mice exposed to different CNTs at different exposure times.

Group	24 h	14 days	29 days	60 days
			**LIVER**	
CTL	6.042 ± 0.76	5.192 ± 0.56	6.129 ± 0.43	5.356 ± 0.53
P-CNTs	5.807 ± 0.53	5.622 ± 0.46	5.857 ± 0.42	5.921 ± 0.49
UP-CNTs	5.156 ± 0.23	5.910 ± 0.28	5.862 ± 0.97	5.194 ± 0.18
FITC-CNTs	5.004 ± 0.41	5.697 ± 0.98	5.613 ± 0.38	6.090 ± 0.49
			**LUNGS**	
CTL	0.704 ± 0.17	0.904 ± 0.53	0.599 ± 0.04	0.607 ± 0.03
P-CNTs	0.723 ± 0.15	0.540 ± 0.05	0.621 ± 0.09	0.578 ± 0.04
UP-CNTs	0.609 ± 0.06	0.616 ± 0.10	0.579 ± 0.012	0.610 ± 0.16
FITC-CNTs	0.535 ± 0.10	0.709 ± 0.03	0.573 ± 0.047	0.703 ± 0.25
			**KIDNEYS**	
CTL	1.371 ± 0.24	1.389 ± 0.09	1.411 ± 0.18	1.351 ± 0.16
P-CNTs	1.256 ± 0.07	1.285 ± 0.09	1.449 ± 0.15	1.479 ± 0.09
UP-CNTs	1.253 ± 0.24	1.283 ± 0.08	1.228 ± 0.12	1.188 ± 0.11
FITC-CNTs	1.276 ± 0.13	1.338 ± 0.04	1.211 ± 0.12	1.643 ± 0.20*
			**SPLEEN**	
CTL	0.551 ± 0.23	0.425 ± 0.13	0.500 ± 0.06	0.397 ± 0.08
P-CNTs	0.435 ± 0.21	0.469 ± 0.24	0.397 ± 0.05	0.416 ± 0.02
UP-CNTs	0.425 ± 0.05	0.466 ± 0.06	0.419 ± 0.15	0.403 ± 0.04
FITC-CNTs	0.428 ± 0.11	0.551 ± 0.14	0.410 ± 0.02	0.407 ± 0.06

Values show media ± S.D. Data were analyzed by Dunnett's test. * Indicated significant differences with the control group.
